# Supplementary material for: Evaluating MINFLUX experimental performance in silico
Source: Nat Commun. 2025 Dec 6;17:246. doi: 10.1038/s41467-025-66952-w (PMC12783130; doi:10.1038/s41467-025-66952-w)
Supplement: Supplementary file 3 — Supplementary Software [file 41467_2025_66952_MOESM3_ESM.zip › SimuFLUX_estimators.pdf]

Estimators used in SimulFLUX

Equations

Calculate normalized probabilities from PSF:

In[∘]:=
```
pnorm[fx_, coordsx_] :=
 Assuming[{L > 0, σq > 0}, With[{px = Map[Simplify[Apply[fx]], coordsx]},
   FullSimplify[px / FullSimplify[Total[px]]]]]
```

Solution to the leased squares problem from Jacobian:

In[∘]:=
```
lsq[J_, Y_] := Assuming[{L > 0, σq > 0, x0 ∈ Reals, y0 ∈ Reals},
  With[{JT = Transpose[J]}, Simplify[Dot[Inverse[Dot[JT, J]], Dot[JT, Y]]]]]
```

Quadratic approximation of donut:

In[∘]:= $f_q[x\_] := A \dfrac{(x - x0)^2}{2 * \sigma^2} + A * b$

In[∘]:=
```
ang2x[tx_, R_] := R * Cos[tx]
ang2y[ty_, R_] := R * Sin[ty]
```

LSQ for 1D quadratic approximation

Experiment : measure ni. Experimental probabilities pi=ni/sum(ni)
Define L2=L/2;

At x0=0, 2 measurements

Linearization of p_i. From Balzarotti 2017, eq. xxx

In[∘]:=
```
pn = pnorm[fq, {{-L2}, {L2}}];
J = FullSimplify[D[pn, {{x0}}] /. x0 → 0];
pv = {p1, p2};
Y = pv - pn /. x0 → 0 // Simplify;
r = lsq[J, Y]
```

Out[∘]=

$$\left\{\frac{(p1 - p2)\left(L2^2 + 2\, b\, \sigma^2\right)}{2\, L2}\right\}$$

At arbitrary x0, for iterative LSQ

In[∘]:=
```
pn = pnorm[f_q, {{-L2}, {L2}}];
J = FullSimplify[D[pn, {{x0}}]];
Y = pv - pn // Simplify;
r2 = lsq[J, Y]
```

Out[∘]=

$$\left\{\frac{\left(L2^2+x0^2+2\,b\,\sigma^2\right)\left(L2^2\,(p1-p2)-2\,L2\,x0+(p1-p2)\left(x0^2+2\,b\,\sigma^2\right)\right)}{2\,L2\left(L2^2-x0^2+2\,b\,\sigma^2\right)}\right\}$$

replace probabilities by difference: d12=p1-p2

In[∘]:=
```
r2r = r2 /. p1 → d12 + p2 // FullSimplify
```

Out[∘]=

$$\left\{\frac{\left(L2^2+x0^2+2\,b\,\sigma^2\right)\left(-2\,L2\,x0+d12\left(L2^2+x0^2+2\,b\,\sigma^2\right)\right)}{2\,L2\left(L2^2-x0^2+2\,b\,\sigma^2\right)}\right\}$$

no background:

In[∘]:=
```
r2r /. b → 0
```

Out[∘]=

$$\left\{\frac{\left(L2^2+x0^2\right)\left(-2\,L2\,x0+d12\left(L2^2+x0^2\right)\right)}{2\,L2\left(L2^2-x0^2\right)}\right\}$$

At arbitrary x0 with center measurement

In[∘]:=
```
pn = pnorm[f_q, {{-L2}, {L2}, {0}}];
J = FullSimplify[D[pn, {{x0}}]];
pv = {p1, p2, p0};
Y = pv - pn // Simplify;
rc = lsq[J, Y] // FullSimplify
```

Out[∘]=

$$\Big\{\Big(\left(2\,L2^2+3\,x0^2+6\,b\,\sigma^2\right)\Big(4\,L2^4\,(p1-p2)+2\,L2^3\,(-3+2\,p0-p1-p2)\,x0+3\,L2\,(4+2\,p0-p1-p2)\,x0^3+24\,b\,L2^2\,(p1-p2)\,\sigma^2+6\,b\,L2\,(-4+2\,p0-p1-p2)\,x0\,\sigma^2-9\,(p1-p2)\left(x0^4-4\,b^2\,\sigma^4\right)\Big)\Big)\Big/\Big(4\left(4\,L2^5+9\,L2\left(x0^2-2\,b\,\sigma^2\right)^2+L2^3\left(-9\,x0^2+24\,b\,\sigma^2\right)\right)\Big)\Big\}$$

Replace : pt = p1 + p2 - 2 p3, d12=p1-p2. Center measurement is measurement 3.

In[∘]:=
```
rcr = FullSimplify[rc /. p0 → 1 / 2 * (p1 + p2 - pt) /. p1 → d12 + p2, L2 > 0]
```

Out[∘]=

$$\Big\{\Big(\left(2\,L2^2+3\,x0^2+6\,b\,\sigma^2\right)\Big(L2\,x0\left(-2\,L2^2\,(3+pt)-3\,(-4+pt)\,x0^2-6\,b\,(4+pt)\,\sigma^2\right)+d12\left(-9\,x0^4+4\left(L2^2+3\,b\,\sigma^2\right)^2\right)\Big)\Big)\Big/\Big(4\left(4\,L2^5+9\,L2\left(x0^2-2\,b\,\sigma^2\right)^2+L2^3\left(-9\,x0^2+24\,b\,\sigma^2\right)\right)\Big)\Big\}$$

In[•]:=
```
rcr /. b → 0 // FullSimplify
```
Out[•]=

$$\left\{\frac{\left(2\,L2^2+3\,x0^2\right)\left(4\,d12\,L2^4-2\,L2^3\,(3+pt)\,x0-3\,L2\,(-4+pt)\,x0^3-9\,d12\,x0^4\right)}{4\left(4\,L2^5-9\,L2^3\,x0^2+9\,L2\,x0^4\right)}\right\}$$

LSQ formulation, find minima directly, 2 point measurement.

In[•]:=
```
pn = pnorm[f_q, {{-L2}, {L2}}] /. b → 0;
s = Solve[
   (-2 * ((p1 - pn[[1]]) * D[pn[[1]], x0] + (p2 - pn[[2]]) * D[pn[[2]], x0])) == 0 /. x0s → x0, {x0}];
rd2 = FullSimplify[s, Assumptions → L2 > 0]
```
Out[•]=

$$\left\{\{x0\to -L2\},\ \{x0\to L2\},\ \left\{x0\to \frac{L2-L2\,\sqrt{1-p1^2+2\,p1\,p2-p2^2}}{p1-p2}\right\},\ \left\{x0\to \frac{L2\left(1+\sqrt{-((-1+p1-p2)\,(1+p1-p2))}\right)}{p1-p2}\right\}\right\}$$

Solutions 1, 2 are maxima, solutions 3, 4 are minima.

In[•]:=
```
FullSimplify[rd2[[{3, 4}]] /. p1 → d12 + p2, L2 > 0]
```
Out[•]=

$$\left\{\left\{x0\to \frac{L2-\sqrt{1-d12^2}\,L2}{d12}\right\},\ \left\{x0\to \frac{\left(1+\sqrt{1-d12^2}\right)L2}{d12}\right\}\right\}$$

Direct solution setting experimental measurements equal to theoretical

3 Point Measurement, background is free fitting parameter

In[•]:=
```
eq = {f_q[-L2] == p1, f_q[L2] == p2, f_q[0] == p0}
```
Out[•]=

$$\left\{A\,b+\frac{A\,(-L2-x0)^2}{2\,\sigma^2}=p1,\ A\,b+\frac{A\,(L2-x0)^2}{2\,\sigma^2}=p2,\ A\,b+\frac{A\,x0^2}{2\,\sigma^2}=p0\right\}$$

In[•]:=
```
sol3 = Solve[eq, {x0, A, b}]
```
Out[•]=

$$\left\{\left\{x0\to \frac{-L2\,p1+L2\,p2}{2\,(2\,p0-p1-p2)},\ A\to \frac{-2\,p0\,\sigma^2+p1\,\sigma^2+p2\,\sigma^2}{L2^2},\ b\to -\frac{L2^2\left(16\,p0^2-8\,p0\,p1+p1^2-8\,p0\,p2-2\,p1\,p2+p2^2\right)}{8\,(2\,p0-p1-p2)^2\,\sigma^2}\right\}\right\}$$

In[•]:=
```
FullSimplify[sol3 /. p0 → 1 / 2 * (p1 + p2 - pt) /. p1 → d12 + p2, L2 > 0]
```

Out[•]=

$$\left\{\left\{x0 \to \frac{d12\ L2}{2\ pt},\ A \to \frac{pt\ \sigma^2}{L2^2},\ b \to -\frac{L2^2\left(d12^2 - 4\ d12\ pt + 4\ pt\ (-2\ p2 + pt)\right)}{8\ pt^2\ \sigma^2}\right\}\right\}$$

2 Point measurement

In[•]:=
```
eq2 = {f_q[-L2] == p1, f_q[L2] == p2} /. b → 0
```

Out[•]=

$$\left\{\frac{A\ (-L2 - x0)^2}{2\ \sigma^2} == p1,\ \frac{A\ (L2 - x0)^2}{2\ \sigma^2} == p2\right\}$$

In[•]:=
```
sol2s = FullSimplify[sol2 = Solve[eq2, {x0, A}], {L2 > 0, p1 > 0, p2 > 0}]
```

Out[•]=

$$\left\{\left\{x0 \to \frac{L2\left(p1 + p2 + 2\sqrt{p1\ p2}\right)}{p1 - p2},\ A \to \frac{\left(p1 + p2 - 2\sqrt{p1\ p2}\right)\sigma^2}{2\ L2^2}\right\},\right.$$
$$\left.\left\{x0 \to L2\left(-1 + \frac{2\sqrt{p1}}{\sqrt{p1} + \sqrt{p2}}\right),\ A \to \frac{\left(p1 + p2 + 2\sqrt{p1\ p2}\right)\sigma^2}{2\ L2^2}\right\}\right\}$$

Compare with MLE

Direct 2 point solution is equal to MLE

In[•]:=
```
xmle1 = -L / 2 + L / (1 + Sqrt[p2] / Sqrt[p1]);
xmle2 = -L / 2 + L / (1 - Sqrt[p2] / Sqrt[p1]);
FullSimplify[xmle2 - x0 /. sol2s[[1]] /. L2 → L / 2, {p1 > 0, p2 > 0}]
```

Out[•]=

0

LSQ for 2 D donut, quadratic approximation

Background set to zero, can be subtracted from measurements. We define pattern positions.

In[ ]:=
```
fD[x_, y_] := A (x - x0)^2 + (y - y0)^2 / (2 σ^2)
R = L / 2;
M3c = {{ang2x[0 π, R], ang2y[0 π, R]},
   {ang2x[2 π / 3, R], ang2y[2 π / 3, R]}, {ang2x[4 π / 3, R], ang2y[4 π / 3, R]}, {0, 0}};
M3 = {{ang2x[0 π, R], ang2y[0 π, R]},
   {ang2x[2 π / 3, R], ang2y[2 π / 3, R]}, {ang2x[4 π / 3, R], ang2y[4 π / 3, R]}};
M4c = {{ang2x[0 π, R], ang2y[0 π, R]}, {ang2x[2 π / 4, R], ang2y[2 π / 4, R]},
   {ang2x[4 π / 4, R], ang2y[4 π / 4, R]}, {ang2x[6 π / 4, R], ang2y[6 π / 4, R]}, {0, 0}};
M4 = {{ang2x[0 π, R], ang2y[0 π, R]}, {ang2x[2 π / 4, R], ang2y[2 π / 4, R]},
   {ang2x[4 π / 4, R], ang2y[4 π / 4, R]}, {ang2x[6 π / 4, R], ang2y[6 π / 4, R]}};
M6c = {{ang2x[0 π, R], ang2y[0 π, R]}, {ang2x[2 π / 6, R], ang2y[2 π / 6, R]},
   {ang2x[4 π / 6, R], ang2y[4 π / 6, R]}, {ang2x[6 π / 6, R], ang2y[6 π / 6, R]},
   {ang2x[8 π / 6, R], ang2y[8 π / 6, R]}, {ang2x[10 π / 6, R], ang2y[10 π / 6, R]}, {0, 0}};
M6 = {{ang2x[0 π, R], ang2y[0 π, R]}, {ang2x[2 π / 6, R], ang2y[2 π / 6, R]},
   {ang2x[4 π / 6, R], ang2y[4 π / 6, R]}, {ang2x[6 π / 6, R], ang2y[6 π / 6, R]},
   {ang2x[8 π / 6, R], ang2y[8 π / 6, R]}, {ang2x[10 π / 6, R], ang2y[10 π / 6, R]}};
```

LSQ for 2D donut around x0==0:

Here we follow Eilers's section 2.2 on page 34. 3 orbit points, no center

In[ ]:=
```
pn = pnorm[fD, M3c];
J = FullSimplify[D[pn, {{x0, y0}}] /. x0 → 0 /. y0 → 0];
pv = {p1, p2, p3, p0};
Y = pv - pn /. x0 → 0 /. y0 → 0 // Simplify;
r = lsq[J, Y]
```

Out[ ]=

$$\left\{-\frac{1}{4} L\,(2\,p1 - p2 - p3),\ -\frac{1}{4}\sqrt{3}\,L\,(p2 - p3)\right\}$$

In[ ]:=
```
-Total[pv * M3c] // FullSimplify
```

Out[ ]=

$$\left\{\frac{1}{4} L\,(-2\,p1 + p2 + p3),\ \frac{1}{4}\sqrt{3}\,L\,(-p2 + p3)\right\}$$

That means: rest = - p·rpattern

Around x0 for iterative LSQ, 4 orbit points + center

In[ ]:=
```
pn = pnorm[f_D, M4c];
J = FullSimplify[D[pn, {{x0, y0}}]];
pv = {p1, p2, p3, p4, p0};
Y = pv - pn // Simplify;
dr = lsq[J, Y];
dr // FullSimplify
```

Out[ ]=

$$\Bigg\{-\frac{1}{4\,L^5 - 30\,L^3\left(x0^2 + y0^2\right) + 100\,L\left(x0^2 + y0^2\right)^2}\left(L^2 + 5\left(x0^2 + y0^2\right)\right)\Big(2\,L^4\,(p1 - p3) + L^3\,(3 - 4\,p0 + p1 + p2 + p3 + p4)\,x0 - 15\,L^2\,y0\,(-p2\,x0 + p4\,x0 + p1\,y0 - p3\,y0) - 5\,L\,(4 + 4\,p0 - p1 - p2 - p3 - p4)\,x0\left(x0^2 + y0^2\right) - 50\left(x0^2 + y0^2\right)\left((p1 - p3)\,x0^2 + 2\,(p2 - p4)\,x0\,y0 + (-p1 + p3)\,y0^2\right)\Big),$$

$$-\frac{1}{4\,L^5 - 30\,L^3\left(x0^2 + y0^2\right) + 100\,L\left(x0^2 + y0^2\right)^2}\left(L^2 + 5\left(x0^2 + y0^2\right)\right)\Big(2\,L^4\,(p2 - p4) + L^3\,(3 - 4\,p0 + p1 + p2 + p3 + p4)\,y0 + 15\,L^2\,x0\,(-p2\,x0 + p4\,x0 + p1\,y0 - p3\,y0) - 5\,L\,(4 + 4\,p0 - p1 - p2 - p3 - p4)\,y0\left(x0^2 + y0^2\right) + 50\left(x0^2 + y0^2\right)\left((p2 - p4)\,x0^2 + 2\,(-p1 + p3)\,x0\,y0 + (-p2 + p4)\,y0^2\right)\Big)\Bigg\}$$

In[ ]:=
```
drs = FullSimplify[dr /. p0 → 1 / 4 * (p1 + p2 + p3 + p4 + ps) /. p2 → p4 + d24 /. p1 → p3 + d13,
   L > 0] // InputForm
```

Out[ ]//InputForm=
```
{-(((L^2 + 5*(x0^2 + y0^2))*(-(x0*(L^3*(-3 + ps) - 15*d24*L^2*y0 + 5*L*(4 + ps)*(x0^2 + y0
   (4*L^5 - 30*L^3*(x0^2 + y0^2) + 100*L*(x0^2 + y0^2)^2)),
 -(((L^2 + 5*(x0^2 + y0^2))*(-(y0*(L^3*(-3 + ps) - 15*d13*L^2*x0 + 5*L*(4 + ps)*(x0^2 + y0
   (4*L^5 - 30*L^3*(x0^2 + y0^2) + 100*L*(x0^2 + y0^2)^2))}
```

Around x0 for iterative LSQ, 4 orbit points

In[∘]:=
```
pn = pnorm[fD, M4];
J = FullSimplify[D[pn, {{x0, y0}}]];
pv = {p1, p2, p3, p4};
Y = pv - pn // Simplify;
dr = lsq[J, Y];
dr // FullSimplify
```

Out[∘]=

$$\left\{-\frac{1}{2\left(L^3-4L\left(x0^2+y0^2\right)\right)}\left(L^2\,(p1-p3)+2\,L\,x0+4\,(p1-p3)\,x0^2+8\,(p2-p4)\,x0\,y0+4\,(-p1+p3)\,y0^2\right)\left(L^2+4\left(x0^2+y0^2\right)\right),\right.$$
$$\left.-\frac{\left((p2-p4)\left(L^2-4\,x0^2\right)+2\,(L+4\,(p1-p3)\,x0)\,y0+4\,(p2-p4)\,y0^2\right)\left(L^2+4\left(x0^2+y0^2\right)\right)}{2\left(L^3-4L\left(x0^2+y0^2\right)\right)}\right\}$$

In[∘]:=
```
drs = FullSimplify[dr /. p2 → p4 + d24 /. p1 → p3 + d13, L > 0] // InputForm
```

Out[∘]//InputForm=
```
{-1/2*((L^2 + 4*(x0^2 + y0^2))*(2*x0*(L + 4*d24*y0) + d13*(L^2 + 4*(x0 - y0)*(x0 + y0))))/
 -1/2*((L^2 + 4*(x0^2 + y0^2))*(2*(L + 4*d13*x0)*y0 + d24*(L^2 - 4*x0^2 + 4*y0^2)))/(L^3 -
```

Around x0 for iterative LSQ, 6 orbit points + center

In[∘]:=
```
pn = pnorm[fD, M6c];
J = FullSimplify[D[pn, {{x0, y0}}]];
pv = {p1, p2, p3, p4, p5, p6, p0};
Y = pv - pn // Simplify;
dr = lsq[J, Y];
dr // FullSimplify // InputForm
```

Out[∘]//InputForm=
```
{-1/12*((3*L^2 + 14*(x0^2 + y0^2))*(-196*(2*p1 + p2 - p3 - 2*p4 - p5 + p6)*x0^4 + 28*x0^3*
     2*x0*(3*L^3*(5 - 6*p0 + p1 + p2 + p3 + p4 + p5 + p6) + 35*Sqrt[3]*L^2*(p2 + p3 - p5 -
     (2*p1 + p2 - p3 - 2*p4 - p5 + p6)*(9*L^4 - 70*L^2*y0^2 + 196*y0^4)))/(9*L^5 - 70*L^3*
 -1/12*((3*L^2 + 14*(x0^2 + y0^2))*(9*Sqrt[3]*L^4*(p2 + p3 - p5 - p6) + 6*L^3*(5 - 6*p0 +
     70*L^2*x0*(Sqrt[3]*(-p2 - p3 + p5 + p6)*x0 + (2*p1 + p2 - p3 - 2*p4 - p5 + p6)*y0) +
     196*(Sqrt[3]*(p2 + p3 - p5 - p6)*x0^4 + 2*(-2*p1 - p2 + p3 + 2*p4 + p5 - p6)*x0^3*y0
   (9*L^5 - 70*L^3*(x0^2 + y0^2) + 196*L*(x0^2 + y0^2)^2)}
```

Around x0 for iterative LSQ, 6 orbit points no center

In[∘]:=
```
pn = pnorm[fD, M6];
J = FullSimplify[D[pn, {{x0, y0}}]];
pv = {p1, p2, p3, p4, p5, p6};
Y = pv - pn // Simplify;
dr = lsq[J, Y];
dr // FullSimplify // InputForm
```

Out[∘]//InputForm=
```
{-1/4*((4*(2*p1 + p2 - p3 - 2*p4 - p5 + p6)*x0^2 + 4*x0*(L + 2*Sqrt[3]*(p2 + p3 - p5 - p6)
    (L^3 - 4*L*(x0^2 + y0^2)), -1/4*(Sqrt[3]*(p2 + p3 - p5 - p6)*(L^4 - 16*x0^4) + 4*(L + 2
     16*(L + 2*(2*p1 + p2 - p3 - 2*p4 - p5 + p6)*x0)*y0^3 + 16*Sqrt[3]*(p2 + p3 - p5 - p6)*
```

Around x0 for iterative LSQ, 3 orbit points + center

In[∘]:=
```
pn = pnorm[fD, M3c];
J = FullSimplify[D[pn, {{x0, y0}}]];
pv = {p1, p2, p3, p0};
Y = pv - pn // Simplify;
dr = lsq[J, Y];
dr // FullSimplify // InputForm
```

Out[∘]//InputForm=
```
{-1/12*((3*L^2 + 16*(x0^2 + y0^2))*(9*L^4*(2*p1 - p2 - p3) + 12*L^3*(2 - 3*p0 + p1 + p2 +
      64*L*(3 + 3*p0 - p1 - p2 - p3)*x0*(x0^2 + y0^2) - 256*(x0^2 + y0^2)*((2*p1 - p2 - p3)
    (9*L^5 - 64*L^3*(x0^2 + y0^2) + 256*L*(x0^2 + y0^2)^2),
  -1/12*((3*L^2 + 16*(x0^2 + y0^2))*(9*Sqrt[3]*L^4*(p2 - p3) + 12*L^3*(2 - 3*p0 + p1 + p2 +
      256*(Sqrt[3]*(p2 - p3)*x0^4 + 2*(-2*p1 + p2 + p3)*x0^3*y0 + 2*(-2*p1 + p2 + p3)*x0*y0
    (9*L^5 - 64*L^3*(x0^2 + y0^2) + 256*L*(x0^2 + y0^2)^2)}
```

Around x0 for iterative LSQ, 3 orbit points no center

In[∘]:=
```
pn = pnorm[fD, M3];
J = FullSimplify[D[pn, {{x0, y0}}]];
pv = {p1, p2, p3};
Y = pv - pn // Simplify;
dr = lsq[J, Y];
dr // FullSimplify // InputForm
```

Out[∘]//InputForm=
```
{-1/4*((4*(2*p1 - p2 - p3)*x0^2 + 4*x0*(L + 2*Sqrt[3]*(p2 - p3)*y0) + (2*p1 - p2 - p3)*(L^
  -1/4*((L^2 + 4*(x0^2 + y0^2))*(Sqrt[3]*L^2*(p2 - p3) + 4*L*y0 - 4*(Sqrt[3]*(p2 - p3)*x0^2
```

LSQ for 2 D Gauss, linear approx. around x=0

Center measurement, N=4

xest = (no + Exp[Ls2])/(Ls2 no)* Sum[$p_i \cdot$ rpat$_i$], Ls2 = $L^2/8\,\sigma^2$ for center measurements

In[ ]:=
```
fG[x_, y_] := A Exp[-((x - x0)^2 + (y - y0)^2)/(2 σ^2)];
pn = pnorm[fG, M4c];
J = FullSimplify[D[pn, {{x0, y0}}] /. x0 → 0 /. y0 → 0];
pv = {p1, p2, p3, p4, p0};
Y = pv - pn /. x0 → 0 /. y0 → 0 // Simplify;
r = lsq[J, Y]
Ls2 = L^2 / 8 / σ^2;
xesteq = (4 + Exp[Ls2]) / Ls2 / 4 * Total[pv * M4c] // FullSimplify;
xesteq - r // Simplify
```

Out[ ]=

$$\left\{\frac{\left(4+e^{\frac{L^2}{8\sigma^2}}\right)(p1-p3)\,\sigma^2}{L},\ \frac{\left(4+e^{\frac{L^2}{8\sigma^2}}\right)(p2-p4)\,\sigma^2}{L}\right\}$$

Out[ ]=

{0, 0}

Center measurement, N = 6

In[ ]:=
```
pn = pnorm[fG, M6c];
J = FullSimplify[D[pn, {{x0, y0}}] /. x0 → 0 /. y0 → 0];
pv = {p1, p2, p3, p4, p5, p6, p0};
Y = pv - pn /. x0 → 0 /. y0 → 0 // Simplify;
r = lsq[J, Y]
Ls2 = L^2 / 8 / σ^2;
xesteq = (6 + Exp[Ls2]) / Ls2 / 6 * Total[pv * M6c] // FullSimplify;
xesteq - r // Simplify
```

Out[ ]=

$$\left\{\frac{\left(6+e^{\frac{L^2}{8\sigma^2}}\right)(2\,p1+p2-p3-2\,p4-p5+p6)\,\sigma^2}{3\,L},\ \frac{\left(6+e^{\frac{L^2}{8\sigma^2}}\right)(p2+p3-p5-p6)\,\sigma^2}{\sqrt{3}\,L}\right\}$$

Out[ ]=

{0, 0}

No center measurement, N=4

xest = 1 / (Ls2) * Sum[$p_i \cdot$ rpat$_i$], Ls2 = L^2 / 8 / σ^2

In[ ]:=
```
pn = pnorm[f_G, M4];
J = FullSimplify[D[pn, {{x0, y0}}] /. x0 → 0 /. y0 → 0];
pv = {p1, p2, p3, p4};
Y = pv - pn /. x0 → 0 /. y0 → 0 // Simplify;
r = lsq[J, Y]
ph = {p1, p2, p3, p4};
xesteq = Total[ph * M4] / Ls2 // FullSimplify;
xesteq - r // Simplify
```

Out[ ]=

$$\left\{\frac{4\,(p1-p3)\,\sigma^2}{L},\ \frac{4\,(p2-p4)\,\sigma^2}{L}\right\}$$

Out[ ]=

{0, 0}

LSQ for 2 D donut, linear approx. around x=0

No measurement, N=4

In[ ]:=

$$f_{do}[x\_,\ y\_] := A\,\frac{(x-x0)^2+(y-y0)^2}{4\,\pi\,\sigma^4}\,\mathrm{Exp}\left[-\frac{(x-x0)^2+(y-y0)^2}{2\,\sigma^2}\right];$$

```
pn = pnorm[f_do, M4c];
J = FullSimplify[D[pn, {{x0, y0}}] /. x0 → 0 /. y0 → 0];
pv = {p1, p2, p3, p4, p0};
Y = pv - pn /. x0 → 0 /. y0 → 0;
r = lsq[J, Y] // FullSimplify
xesteq = 1 / (L^2 / (8 σ^2) - 1) * Total[pv * M4c] // FullSimplify;
xesteq - r // Simplify
```

Out[ ]=

$$\left\{\frac{4\,L\,(p1-p3)\,\sigma^2}{L^2-8\,\sigma^2},\ \frac{4\,L\,(p2-p4)\,\sigma^2}{L^2-8\,\sigma^2}\right\}$$

Out[ ]=

{0, 0}

No measurement, N=4

In[ ]:=
```
fB[x_, y_] := A 4 Exp[1] Log[2] ((x - x0)^2 + (y - y0)^2)/fwhm^2 Exp[-4 Log[2] ((x - x0)^2 + (y - y0)^2)/fwhm^2];
pn = pnorm[fB, M4c];
J = FullSimplify[D[pn, {{x0, y0}}] /. x0 → 0 /. y0 → 0];
pv = {p1, p2, p3, p4, p0};
Y = pv - pn /. x0 → 0 /. y0 → 0;
r = lsq[J, Y] // FullSimplify
xesteq = 1 / (Log[2 ] L^2 / (fwhm^2) - 1) * Total[pv * M4c] // FullSimplify;
xesteq - r // Simplify
```

Out[ ]=

$$\left\{\frac{\text{fwhm}^2\, L\, (p1 - p3)}{-2\,\text{fwhm}^2 + L^2\, \text{Log}[4]}, \frac{\text{fwhm}^2\, L\, (p2 - p4)}{-2\,\text{fwhm}^2 + L^2\, \text{Log}[4]}\right\}$$

Out[ ]=

{0, 0}

In[ ]:=

Quadratic approximation of donut

In[ ]:=
```
fr[r_] := A (r)^2/(4 π σ^4) Exp[-r^2/(2 σ^2)];
```

In[ ]:=
```
Series[fr[x], {x, 0, 2}]
```

Out[ ]=

$$\frac{A\, x^2}{4\, \pi\, \sigma^4} + O[x]^3$$
